# Genomic Analysis of Novel Poxvirus Brazilian Porcupinepox Virus, Brazil, 2019

**DOI:** 10.3201/eid2704.203818

**Published:** 2021-04

**Authors:** Aline S. Hora, Sueli A. Taniwaki, Nathana B. Martins, Nataly N.R. Pinto, André E. Schlemper, André L.Q. Santos, Matias P.J. Szabó, Paulo E. Brandão

**Affiliations:** Federal University of Uberlândia, Minas Gerais, Brazil (A.S. Hora, N.B. Martins, N.N.R. Pinto, A.E. Schlemper, A.L.Q. Santos, M.P.J. Szabó);; University of São Paulo, São Paulo, Brazil (S.A. Taniwaki, P.E. Brandão)

**Keywords:** novel poxvirus, viral disease, emerging infectious, viruses, zoonoses, Brazil, Poxviridae, Chordopoxvirinae, Brazilian porcupinepox virus

## Abstract

We obtained the complete sequence of a novel poxvirus, tentatively named Brazilian porcupinepox virus, from a wild porcupine (*Coendou prehensilis*) in Brazil that had skin and internal lesions characteristic of poxvirus infection. The impact of this lethal poxvirus on the survival of this species and its potential zoonotic importance remain to be investigated.

Poxviruses are among the best known and most feared viruses (*1*); the *Poxviridae* family includes several viruses of veterinary and medical relevance, some of them zoonotic. Emergence and reemergence of poxviruses is frequently observed (*2*–*4*). We report a systemic and lethal poxvirus infection in a wild porcupine and further characterize the virus through genomic analysis.

## The Study

**Figure 1:**
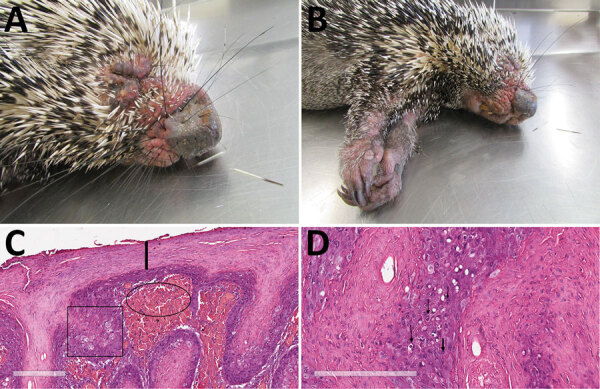
Photographs and histopathology of Brazilian porcupine (*Coendou prehensilis)* with novel poxvirus tentatively named Brazilian porcupinepox virus, Brazil, 2019. A) Severely swollen and erythematous skin of the eyelids, nasal region, and around oral cavity. B) Severely swollen skin of the forelimbs. C) Histopathologic examination of skin. Marked epidermal hyperplasia and swollen epithelial cells with foci of ballooning degeneration are marked with the square, and parakeratotic hyperkeratosis is indicated by the line. Dermal hemorrhage at the dermal–epidermal junction is indicated with the oval. Hematoxylin and eosin stain. Scale bar indicates 200 μm. D) Histopathologic examination of skin. Cytoplasm of several epithelial cells of epidermis with round eosinophilic inclusions is indicated by arrows. Hematoxylin and eosin stain. Scale bar indicates 200 μm.

In March 2019, a free-ranging adult male Brazilian porcupine (*Coendou prehensilis*) in good bodily condition was captured near a park in the urban area of Uberlândia in the state of Minas Gerais in southeastern Brazil and was then referred for veterinary clinical care at the Federal University of Uberlândia. The animal had multifocal skin edema and erythema, especially on the eyelid and muzzle (Figure 1, panel A), extremity of limbs (Figure 1, panel B), and genital areas, and a penetrating skin lesion on the lateral face of the right limb near the elbow joint. We observed purulent nasal and ocular secretion. After 4 days of supportive treatment, the porcupine died and was subjected to a full necropsy for histopathologic evaluation and to collect samples for molecular investigation.

On macroscopic examination, the spleen was enlarged, lungs were turgid, and the liver was pale and greyish. Standard histopathologic sections were cut from formalin-fixed, paraffin-embedded skin and organ samples and stained with hematoxylin and eosin. Histologic examination of the skin revealed several common alterations, irrespective of the location of the sample (Figure 1, panels C and D). Epidermis exhibited marked hyperplasia, parakeratotic hyperkeratosis, and moderate acantholysis. Epidermal cells were swollen, with foci of ballooning degeneration, and the cytoplasm of scattered epithelial cells contained round eosinophilic inclusions of varying size. No intranuclear inclusions were found. We also observed ulcerated epidermis with eosinophilic, amorphous keratinaceous crusts, necrosis, and numerous degenerated granulocytes. Dermal lesions included hemorrhage at the dermal–epidermal junction, severe edema, necrotic areas, and mixed inflammatory infiltrate that extended into the deep dermis. Venous blood congestion was observed in the kidneys, liver, spleen, and lungs. Hepatocytes evidenced moderate degeneration, whereas emphysema and pneumonitis were observed in the lungs.

We extracted total DNA from lesioned eyelid skin, spleen, and liver samples and subjected to a pan-pox universal PCR assay (*5*). All samples resulted in amplicons with low–GC content poxvirus primers targeting a region of the putative metalloproteinase gene. The amplicon from lesioned eyelid skin was submitted for Sanger sequencing (GenBank accession no. MK944278), and total DNA from this lesion was submitted to full-genome sequencing using the Illumina NextSeq platform (Illumina, https://www.illumina.com). We performed viral particle enrichment and next-generation sequencing (Appendix). A total of 71,507,840 pairs of 151-bp reads were obtained after raw data quality control using CLC Genomics Workbench 11 (QIAGEN, https://www.qiagen.com). The resulting paired-end reads were de novo assembled in CLC Genomics Workbench 11 (QIAGEN) with default parameters, resulting in a 144,504-nt genome (GenBank accession no. MN692191) with average coverage of 230.41x.

We annotated the genome (Appendix). Of 133 open reading frames (ORFs) found, only 2 (117 and 129) have no equivalents in other poxviruses, ORFs situated in the middle region of the genome encode proteins related to virion morphogenesis, the structure of virus particles, and viral DNA and RNA metabolism. We also identified the minimum essential chordopoxvirus genome, 49 genes conserved between highly diverged poxviruses families, and 41 genes conserved in chordopoxviruses (*6*). ORFs related to host range, immunomodulation, and virulence were observed in the extremities of the genome.

**Figure 2:**
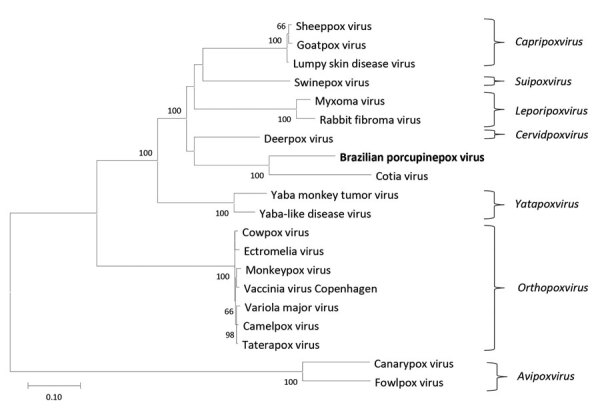
Phylogenetic tree constructed in genomic analysis of novel poxvirus Brazilian porcupinepox virus, Brazil, 2019 (boldface). Tree constructed by using the maximum-likelihood method and Jones–Taylor–Thornton model (*7*) with frequency model for amino acid sequence alignments of the RNA polymerase subunit RPO147, RNA polymerase subunit RPO132, RNA polymerase–associated RAP94, mRNA capping enzyme large subunit, virion major core protein P4a, early transcription factor VETFL, nucleoside-triphosphatase, DNA polymerase, and DNA topoisomerase I genes of selected strains representing different genera of chordopoxvirus with low GC contents and their respective genera. The numbers next to each node represent the values of 1,000 bootstrap repetitions, and only those >50% are shown. Evolutionary analyses were conducted in MEGA X (*8*). GenBank accession numbers are as follows: Brazilian porcupinepox virus, MK944278.1; camelpox virus, AY009089.1; canarypox virus, NC005309.1; Cotia virus, KM595078.1; cowpox virus, DQ437593.1; deerpox virus, AY689437.1; ectromelia virus, NC004105.1; fowlpox virus, NC002188.1; goatpox virus, MH381810.1; lumpy skin disease virus, NC003027.1; monkeypox virus, DQ011157.1; myxoma virus, NC001132.2; rabbit fibroma virus, NC001266.1; sheeppox virus, NC004002.1; swinepox virus, NC003389.1; taterapox virus, NC008291.1; vaccinia virus, M35027.1; variola major virus, L22579.1; Yaba monkey tumor virus, NC005179.1; Yaba-like disease virus, NC002642.1. Scale bar represents number of substitutions per site.

Alignments of 9 amino acid sequences corresponding to 9 conserved genes located in the central region were concatenated (7,956 aa) compiled from this genome with homologous sequences from different genera of chordopoxviruses with low GC content. Thus, we constructed a phylogenetic tree by using the maximum-likelihood method and Jones–Taylor–Thornton model (*7*) in MEGA X software (*8*) with the frequency matrix model (Figure 2). A nucleotide tree with complete genomes was also constructed (Appendix Figure 1).

Using the 9 concatenated sequences corresponding to the conserved central region of MN692191 and other known chordopoxviruses, we obtained nucleotide identities ranged from 69.9% to 85.2% (Appendix Figure 2) and the amino acid identities ranged from 57.7% to 78.8% (Appendix Figure 3). The highest identity was observed between MN692191 and Cotia virus (CoTV) in clusters in the phylogenetic trees. In the 1960s, CoTV was isolated in Brazil from sentinel suckling mice (*9*); the natural host remains unknown and CoTV remains unclassified despite attempts to place it in a new genus of *Poxviridae* (*10*). According to the International Committee on Taxonomy of Viruses (*11*), isolates within a species exhibit >98% nucleotide identity. CoTV and MN692191 exhibit 85.2% of nucleotide identity and thus are distinct species.

We estimated maximum-likelihood distances for this region of conserved nucleotides; the distance between CoTV and MN692191 was 0.156. MN692191 belongs to a main clade that includes *Capripoxvirus, Suipoxvirus, Leporipoxvirus, Cervidpoxvirus*, and *Yatapoxvirus* (clade CSLCY). Distances ranging from 0.177 to 0.210 were observed between MN692191 and species from different genera of the CSLCY clade. In species from the same genus of this clade, distance ranged from 0.006 to 0.052, which suggests that MN692191 and CoTV (maximum-likelihood distance 0.156) do not belong to the same genus.

## Conclusions

This comprehensive phylogenetic analysis supports the classification of MN692191 into a new genus in the family *Poxviridae*, subfamily *Chordopoxvirinae*. Because the virus described in this study is distinct from previously identified viruses, we propose the tentative species name Brazilian porcupinepox virus (BPoPV), according to recommendations for nomenclature of poxvirus species of the International Committee on Taxonomy of Viruses (*11*).

Wildlife veterinarians in Brazil have observed free-ranging porcupines exhibiting clinical signs compatible with those described in this study (B.S.S. Petri, CRAS Parque Ecológico do Tietê- São Paulo, pers. comm., 2019 Sep 16; I.S. Barbosa, CETAS–Goiânia, pers. comm., 2020 Jan 6) have been observed. In 2019, of 13 of these porcupine specimens reported, only 3 had fully recovered from clinical symptoms, demonstrating that this virus might be a common pathogen for this species and could have consequences for its conservation.

Brazilian porcupines have a greater distribution in Brazil but are found in 10 other countries in Latin America (*12*). This species is found mainly in forest environments (*12*) and can be observed in forest fragments in urban areas, as was the case for the specimen in this study. Housing construction nearer to forested areas has led to this porcupine sometimes being hunted for meat (*13*), which leads to human exposure to the pathogens hosted by this species.

The genus *Orthopoxvirus* includes the best-known zoonotic poxvirus species, such as cowpox, monkeypox, and vaccinia viruses (*14*); however, the zoonotic poxvirus is not restricted to this genus. The genera *Parapoxvirus* and *Yatapoxvirus* also include viral species of zoonotic importance (*15*). Furthermore, chordopoxviruses are often described as emerging zoonoses. Contact between Brazilian porcupines and humans, because of anthropized forested areas and the porcupines’ broad geographic distribution and presence in urban areas, raises concerns about the zoonotic potential of BPoPV, which remains to be investigated.

In summary, our description of this novel poxvirus contributes to knowledge of viral diversity and pathogenicity of poxviruses. Some chordopoxviruses are capable of infecting multiple animal species, whereas others have a restricted host spectrum (*14*,*15*). The infection capability of BPoPV is a crucial aspect for further study. 

Appendix Additional information about genomic analysis of novel poxvirus Brazilian porcupinepox virus, Brazil, 2019.
