# Supplementary material for: Genomic Analysis of Novel Poxvirus Brazilian Porcupinepox Virus, Brazil, 2019
Source: Emerg Infect Dis. 2021 Apr;27(4):1177–80. doi: 10.3201/eid2704.203818 (PMC8007330; doi:10.3201/eid2704.203818)
Supplement: Appendix — Additional information about genomic analysis of novel poxvirus Brazilian porcupinepox virus, Brazil, 2019. [file 20-3818-Techapp-s1.pdf]

Article DOI: https://doi.org/10.3201/eid2704.203818

Genomic Analysis of Novel Poxvirus Brazilian Porcupinepox Virus, Brazil, 2019

Appendix

Summary of Viral Enrichment to Next-Generation Sequencing

For viral particle enrichment before next-generation sequencing, the eyelid lesion was cut into small pieces, added to 1mL of TE 1x (pH 8.0), and frozen and thawed twice. The sample was clarified at 16,000xg/3min and filtrate at 0.45um syringe filter. Nuclease treatment was performed in a final volume of 300uL with 50U of Benzonase Nuclease (Sigma-Aldrich, https://www.sigmaaldrich.com), 20U of Turbo DNase (ThermoFisher Scientific, https://www.thermofisher.com), 6U of RNase Cocktail Enzyme Mix, and 1x Turbo DNase Buffer. Reaction was incubated at 37°C for 1h and DNA extraction was performed immediately by using DNeasy Blood and Tissue kit (QIAGEN, https://www.qiagen.com).

Summary of Genome Annotation

We annotated the genome by using a combination of 3 methods: Genome Annotation Transfer Utility (GATU) (*1*), GeneMarkS-2 (*2*), and Vgas (*3*). The open reading frames >50 aa with ≤25% overlap with neighboring genes were initially selected. Two proteins were considered similar if BLAST search resulted an e-value $\leq 10^{-5}$ and maximal scoring pair alignment in the BLASTp searches covered ≥40% of the longer protein.

References

<jrn>1. Tcherepanov V, Ehlers A, Upton C. Genome Annotation Transfer Utility (GATU): rapid annotation of viral genomes using a closely related reference genome. BMC Genomics. 2006;7:150. PubMed https://doi.org/10.1186/1471-2164-7-150</jrn>

<jrn>2. Lomsadze A, Gemayel K, Tang S, Borodovsky M. Modeling leaderless transcription and atypical genes results in more accurate gene prediction in prokaryotes. Genome Res. 2018;28:1079–89. PubMed https://doi.org/10.1101/gr.230615.117</jrn>

<jrn>3. Zhang KY, Gao YZ, Du MZ, Liu S, Dong C, Guo FB. Vgas: a viral genome annotation system. Front Microbiol. 2019;10:184. PubMed https://doi.org/10.3389/fmicb.2019.00184</jrn>

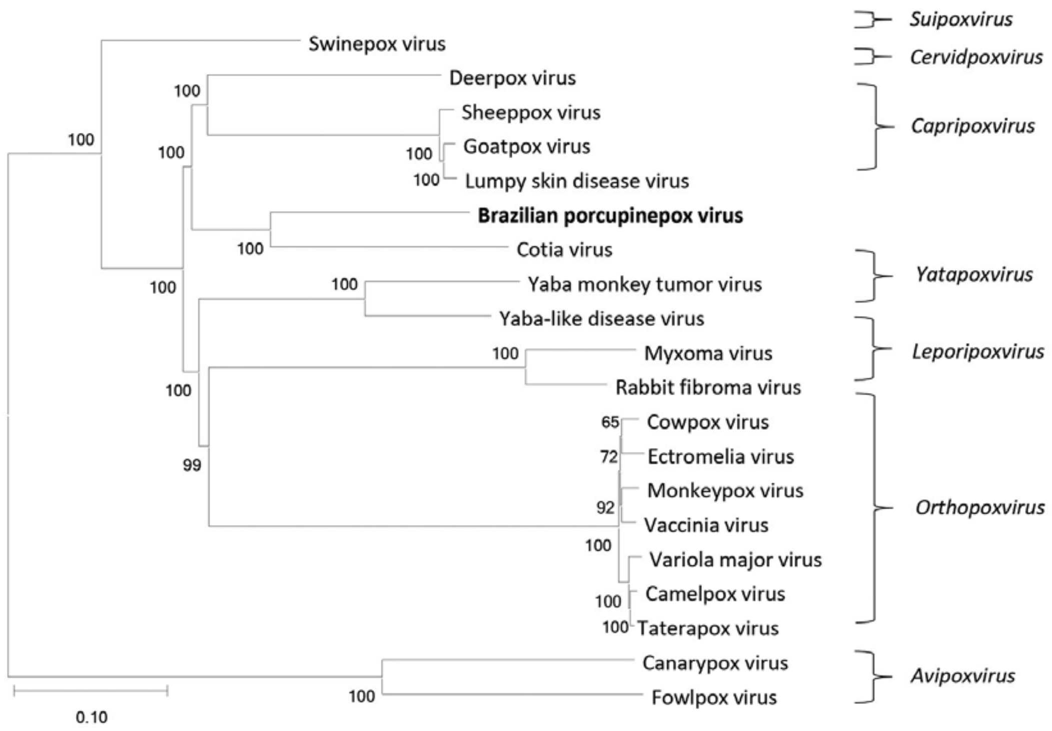

Appendix Figure 1. Phylogenetic tree constructed in genomic analysis of novel poxvirus Brazilian porcupinepox virus, Brazil, 2019. Tree constructed by using the Neighbor-Joining method and Jukes-Cantor model for complete genomes of selected strains representing different genera of chordopoxviruses with low GC contents. The numbers next to each node represent the values of 1,000 bootstrap repetitions, and only those >50% are shown. Evolutionary analyses were conducted in MAFFT (https://mafft.cbrc.jp). GenBank accession numbers are as follows: Brazilian porcupinepox virus, MK944278.1; Camelpox virus, AY009089.1; Canarypox virus, NC005309.1; Cotia virus, KM595078.1; Cowpox virus, DQ437593.1; Deerpox virus, AY689437.1; Ectromelia virus, NC004105.1; Fowlpox virus, NC002188.1; Goatpox virus, MH381810.1; Lumpy skin disease virus, NC003027.1; Monkeypox virus, DQ011157.1; Myxoma virus, NC001132.2; Rabbit fibroma virus, NC001266.1; Sheeppox virus, NC004002.1; Swinepox virus, NC003389.1; Taterapox virus, NC008291.1; Vaccinia virus, M35027.1; Variola major virus, L22579.1; Yaba monkey tumor virus, NC005179.1; Yaba-like disease virus, NC002642.1.

	Brazilian porcupinepox virus	Canarypox virus	Fowlpox virus	Swinepox virus	Cotia virus	Lumpy skin disease virus	Deerpox virus	Cowpox virus	Ectromelia virus	Monkeypox virus	Vaccinia virus	Camelpox virus	Taterapox virus	Variola major virus	Myxoma virus	Rabbit fibroma virus	Yaba monkey tumor virus	Yaba-like disease virus	Sheeppox virus	Goatpox virus
Brazilian porcupinepox virus	ID																			
Canarypox virus	69.9	ID																		
Fowlpox virus	70.2	87.6	ID																	
Swinepox virus	82.9	70.1	70	ID																
Cotia virus	**85.2**	69.3	69.7	82.5	ID															
Lumpy skin disease virus	82.9	70.3	70.3	86.3	82.5	ID														
Deerpox virus	83.4	70.1	70.2	85.9	82	85.7	ID													
Cowpox virus	77.5	69.9	69.7	79	76.5	79	79.6	ID												
Ectromelia virus	77.4	69.8	69.5	78.9	76.4	78.8	79.4	99.1	ID											
Monkeypox virus	77.4	69.8	69.5	79	76.4	78.9	79.4	99	98.9	ID										
Vaccinia virus Copenhagen	77.4	69.9	69.6	79	76.5	78.9	79.4	99.2	99	99.1	ID									
Camelpox virus	77.4	69.9	69.6	78.9	76.4	78.9	79.4	99.1	99	99	99.2	ID								
Taterapox virus	77.3	69.8	69.5	78.9	76.4	78.9	79.3	99.2	99	99.1	99.2	99.5	ID							
Variola major virus	77.3	69.8	69.6	78.9	76.3	78.8	79.3	98.9	98.8	98.8	98.9	99.2	99.2	ID						
Myxoma virus	81.4	69.5	69.4	83.3	80	83.5	85.3	78.1	78	77.9	78	78	78	77.9	ID					
Rabbit fibroma virus	81.5	69.4	69.3	83.2	80	83.5	85.4	78.1	78	78	78	78	78	77.9	96.4	ID				
Yaba monkey tumor virus	81	69.9	70.1	82.8	80.2	83.4	84.1	78.7	78.6	78.7	78.7	78.6	78.7	78.7	82	81.9	ID			
Yaba-like disease virus	81.2	70.4	70.3	83.5	80.2	84.2	84.7	79.4	79.2	79.3	79.3	79.3	79.3	79.2	82.4	82.4	94.5	ID		
Sheeppox virus	82.8	70.4	70.2	86.3	82.5	99.4	85.6	78.9	78.7	78.8	78.8	78.8	78.7	78.8	83.5	83.4	83.4	84.2	ID	
Goatpox virus	82.8	70.4	70.3	86.3	82.5	99.3	85.6	79	78.8	78.9	78.9	78.8	78.8	78.8	83.5	83.5	83.4	84.2	99.1	ID

Appendix Figure 2. Sequence identity matrix in genomic analysis of novel poxvirus Brazilian porcupinepox virus, Brazil, 2019. Matrix shows nucleotides identity (%) of the RNA polymerase subunit RPO147, RNA polymerase subunit RPO132, RNA polymerase-associated RAP94, mRNA capping enzyme large subunit, virion major core protein P4a, early transcription factor VETFL, nucleoside triphosphatase (NTPase), DNA polymerase, and DNA topoisomerase I genes of selected strains representing different genera of chordopoxviruses with low GC contents and their respective genera. In bold, the highest identity related to Brazilian porcupinepox virus. GenBank accession numbers are as follows: Brazilian porcupinepox virus, MK944278.1; Camelpox virus, AY009089.1; Canarypox virus, NC005309.1; Cotia virus, KM595078.1; Cowpox virus, DQ437593.1; Deerpox virus, AY689437.1; Ectromelia virus, NC004105.1; Fowlpox virus, NC002188.1; Goatpox virus, MH381810.1; Lumpy skin disease virus, NC003027.1; Monkeypox virus, DQ011157.1; Myxoma virus, NC001132.2; Rabbit fibroma virus, NC001266.1; Sheeppox virus, NC004002.1; Swinepox virus, NC003389.1; Taterapox virus, NC008291.1; Vaccinia virus, M35027.1; Variola major virus, L22579.1; Yaba monkey tumor virus, NC005179.1; Yaba-like disease virus, NC002642.1.

	Brazilian porcupinepox virus	Canarypox virus	Fowlpox virus	Swinepox virus	Cotia virus	Lumpy skin disease virus	Deerpox virus	Cowpox virus	Ectromelia virus	Monkeypox virus	Vaccinia virus	Camelpox virus	Taterapox virus	Variola major virus	Myxoma virus	Rabbit fibroma virus	Yaba monkey tumor virus	Yaba-like disease virus	Sheeppox virus	Goatpox virus
Brazilian porcupinepox virus	ID																			
Canarypox virus	57.7	ID																		
Fowlpox virus	58.1	81.7	ID																	
Swinepox virus	75.6	58.4	58.4	ID																
Cotia virus	**78.8**	56.9	57.3	73.1	ID															
Lumpy skin disease virus	75.2	58.4	58.5	79.0	73.0	ID														
Deerpox virus	76.4	58.1	58.4	80.5	73.8	80.0	ID													
Cowpox virus	67.5	57.5	57.5	69.6	65.9	70.0	70.7	ID												
Ectromelia virus	67.3	57.5	57.4	69.5	65.8	69.7	70.5	98.6	ID											
Monkeypox virus	67.4	57.4	57.2	69.5	65.8	69.8	70.5	98.3	98.2	ID										
Vaccinia virus	67.4	57.6	57.4	69.6	65.8	69.8	70.6	98.7	98.4	98.6	ID									
Camelpox virus	67.3	57.4	57.4	69.4	65.7	69.8	70.5	98.6	98.3	98.4	98.7	ID								
Taterapox virus	67.2	57.4	57.3	69.5	65.7	69.7	70.4	98.7	98.5	98.6	98.7	99.2	ID							
Variola major virus	67.3	57.4	57.3	69.5	65.7	69.7	70.4	98.3	98.1	98.1	98.3	98.7	98.8	ID						
Myxoma virus	73.9	57.7	57.8	77.0	71.6	77.5	77.8	69.2	68.9	68.9	69.0	69.0	69.0	68.9	ID					
Rabbit fibroma virus	73.8	57.5	57.8	76.8	71.6	77.0	77.7	69.1	68.9	69.0	69.0	69.0	69.0	68.9	94.3	ID				
Yaba monkey tumor virus	72.7	57.8	58.1	75.5	71.4	76.5	77.5	70.0	69.8	69.9	70.0	69.9	69.9	69.9	74.7	74.6	ID			
Yaba-like disease virus	73.1	58.2	58.3	76.3	71.5	77.5	78.3	70.9	70.6	70.8	70.8	70.7	70.6	70.5	75.3	75.4	91.3	ID		
Sheeppox virus	75.1	58.3	58.5	78.9	73.0	98.9	79.9	69.8	69.5	69.6	69.6	69.6	69.5	69.6	77.3	76.9	76.5	77.6	ID	
Goatpox virus	7.1	58.4	58.5	78.9	73.0	98.8	79.9	69.8	69.5	69.7	69.7	69.6	69.6	69.6	77.4	76.9	76.5	77.5	98.6	ID

Appendix Figure 3. Sequence identity matrix in genomic analysis of novel poxvirus Brazilian porcupinepox virus, Brazil, 2019. Matrix shows the amino acid identity (%) of the RNA polymerase subunit RPO147, RNA polymerase subunit RPO132, RNA polymerase-associated RAP94, mRNA capping enzyme large subunit, virion major core protein P4a, early transcription factor VETFL, nucleoside triphosphatase (NTPase), DNA polymerase, and DNA topoisomerase I genes of selected strains representing different genera of chordopoxviruses with low GC contents and their respective genera. The highest identity related to Brazilian porcupinepox virus is shown in boldface. GenBank accession number are as follows: Brazilian porcupinepox virus, MK944278.1; Camelpox virus, AY009089.1; Canarypox virus, NC005309.1; Cotia virus, KM595078.1; Cowpox virus, DQ437593.1; Deerpox virus, AY689437.1; Ectromelia virus, NC004105.1; Fowlpox virus, NC002188.1; Goatpox virus, MH381810.1; Lumpy skin disease virus, NC003027.1; Monkeypox virus, DQ011157.1; Myxoma virus, NC001132.2; Rabbit fibroma virus, NC001266.1; Sheeppox virus, NC004002.1; Swinepox virus, NC003389.1; Taterapox virus, NC008291.1; Vaccinia virus, M35027.1; Variola major virus, L22579.1; Yaba monkey tumor virus, NC005179.1; Yaba-like disease virus, NC002642.1.
